# Record electron self-cooling in cold-electron bolometers with a hybrid superconductor-ferromagnetic nanoabsorber and traps

**DOI:** 10.1038/s41598-020-78869-z

**Published:** 2020-12-15

**Authors:** A. V. Gordeeva, A. L. Pankratov, N. G. Pugach, A. S. Vasenko, V. O. Zbrozhek, A. V. Blagodatkin, D. A. Pimanov, L. S. Kuzmin

**Affiliations:** 1 Nizhny Novgorod State Technical University n.a. R.E. Alekseev, GSP-41, Nizhny Novgorod, 603950 Russia; 2 Chalmers University of Technology, 41296 Göteborg, Sweden; 3 Institute for Physics of Microstructures of RAS, GSP-105, Nizhny Novgorod, 603950 Russia; 4 Skobeltsyn Institute of Nuclear Physics Lomonosov Moscow State University, Leninskie Gory 1-2, Moscow, 119991 Russia; 5 National Research University Higher School of Economics, Moscow, 101000 Russia; 6 Donostia International Physics Center (DIPC), 20018 San Sebastián/Donostia, Basque Country Spain

**Keywords:** Superconducting devices, Superconducting properties and materials

## Abstract

The Cosmic Microwave Background (CMB) radiation is the only observable that allows studying the earliest stage of the Universe. Radioastronomy instruments for CMB investigation require low working temperatures around 100 mK to get the necessary sensitivity. On-chip electron cooling of receivers is a pathway for future space missions due to problems of dilution fridges at low gravity. Here, we demonstrate experimentally that in a Cold-Electron Bolometer (CEB) a theoretical limit of electron cooling down to 65 mK from phonon temperature of 300 mK can be reached. It is possible due to effective withdrawing of hot electrons from the tunnel barrier by double stock, special traps and suppression of Andreev Joule heating in hybrid Al/Fe normal nanoabsorber.

The Cosmic Microwave Background (CMB) radiation is the most ancient electro-magnetic source of information about the history of our Universe. Thanks to modern technologies, we can measure the temperature and polarization of the photons generated about 370,000 years after the Big Bang (which corresponds to the cosmological red shift $Z = 10^{3}$) with high precision. Moreover, these photons might have imprints from even earlier times, left by the primordial gravitational waves when the Universe was about $10^{-37}\ \mathrm{s}$ old^1–3^. In a typical cosmological experiment, the radiation, travelled to us from the sphere of a radius of the order of $10^{-23}\ \mathrm{km}$, is concentrated into a nanoabsorber with high responsivity.

Several models predict that the primordial gravitational waves can be detected in the form of a polarized signal in the CMB, the so-called “B-modes”, which have magnitude much below ${0.1}\ \upmu \mathrm{K}$. To achieve the required sensitivity, radioastronomy instruments must be cooled below 100 mK^4–6^. Such temperatures are a serious challenge for space applications since the conventional closed-cycle dilution refrigerators require gravity for their operation. In particular, the open-cycle dilution refrigerator (OCDR) aboard the Planck satellite^7^ operated in zero gravity by ejecting the $^3\hbox {He}/^4\hbox {He}$ mixture into space. The lifetime of this OCDR with $0.1\ \upmu \mathrm{W}$ of cooling power at 100 mK was about two years. Instruments aboard future space missions such as SPICA and COrE require higher cooling powers of the order 1–3$\ \upmu \mathrm{W}$ at 100 mK and longer operating times of about five years^8^. The development of a new gravity-independent closed-cycle dilution refrigerator is an attempt to solve this complicated task^9^.

An alternative approach, motivated by the widespread use of 300 mK range $^3$He cryostats for space applications, is an on-chip electron cooling of receivers using NIS (Normal metal–Insulator–Superconductor) tunnel junctions^10^. Biased below the superconducting gap, the NIS junctions can cool the conduction electrons in normal metals below the phonon temperature owing to the selective tunneling of hot electrons induced by the energy gap in the S electrode^11,12^. In metals at low temperatures, the electron–phonon relaxation becomes orders of magnitude slower than the electron–electron relaxation^13,14^. As a result, the electron and phonon subsystems can co-exist with different but well-defined temperatures.

NIS cooling was used to create micro-refrigerator platforms representing a membrane with several NIS junctions around its perimeter^15–19^. It was expected that the cooling power of such a platform would be significant to cool a nano-sized detector, placed on it, below 300 mK. In practice, the cooling efficiency quickly drops with temperature, when a phonon system needs to be cooled through an electron system. It happens due to weak electron–phonon coupling (decreasing with temperature as $\sim T^4$)^20^ and correspondingly low electron–phonon thermal conductance.

A more efficient solution could be to cool an absorber of a detector only. The maximum efficiency would be reached in the case when the absorber is the electron gas of a normal metal. This concept is fully realized in the Cold-Electron Bolometer (CEB)^21–27^. There, radiation is absorbed by the electron subsystem of a normal metal surrounded by two tunnel junctions, forming a symmetric SINIS structure (Fig. 1a). Such a structure has twice more efficient electron cooling than a system with one NIS junction^28^.

The theory predicts, that the electron temperature can be reduced from 300 mK to around 50 mK^29^ in hybrid superconductive/ferromagnetic (S/F) structures^30^. In experiments, the electron cooling by 200 mK at the base temperature 300 mK was demonstrated in several systems with Cu absorber^28,31,32^, and down to 82 mK with a composite AlMn absorber^17^. There are several reasons, given in “Discussion” section, why in practice it is difficult to reach the theoretical minimum.

Here, we report a record electron cooling from 300 to 65 mK in the samples with a hybrid S/F absorber. It was done by accomplishing the following tasks: (1) the double stock (described in the next section); (2) thin normal metal traps under massive superconductors were introduced for effective removal of the heat of relaxed quasiparticles from the absorber; (3) the Andreev current was suppressed by the hybrid S/F nanoabsorber.

Below, we present three types of CEBs with different cooling efficiencies, focusing mostly on the third design, as the most effective. For the design **A**^26^ about 30% of the heat, removed through the NIS junctions, returns back to the absorber. In the designs **B**^27^ and **C**, we decrease the returning heat to 6% and 0.5%, respectively. Thus, we have nearly approached the theoretical minimum for the electron cooling in SINIS structures^29^, which makes CEB a promising candidate for prospective receivers on future space missions.

**Figure 1:**
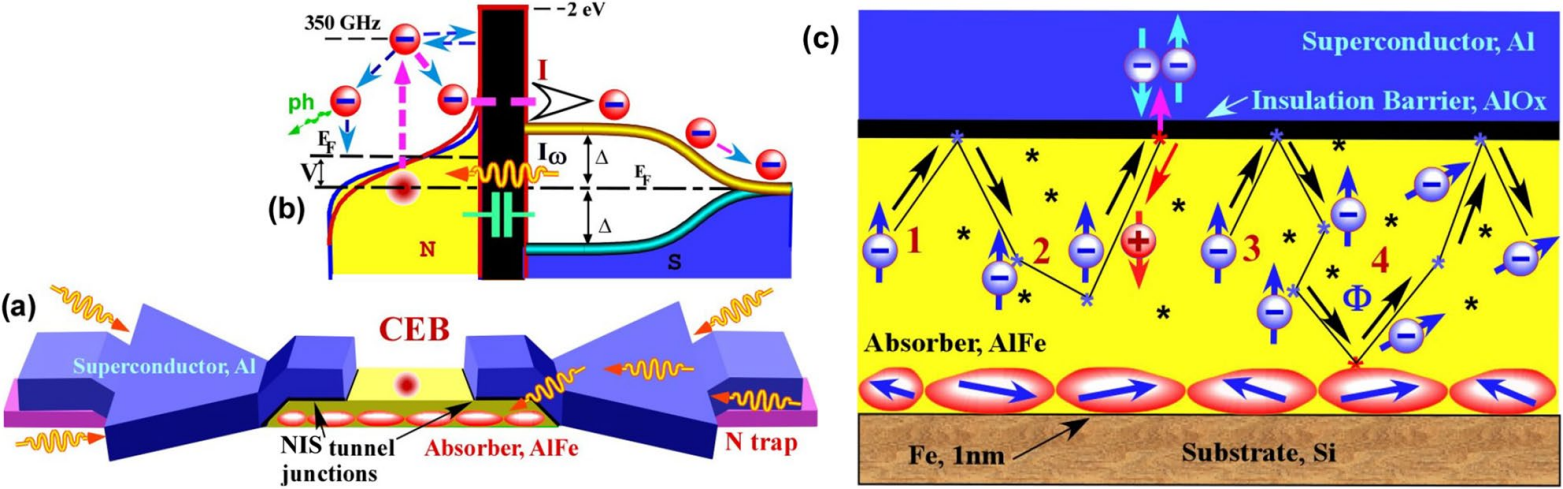
(**a**) 3D scheme of CEB. The absorber is shown in yellow (Al) with red grains (Fe). The superconductivity of Al is suppressed by Fe grains. Blue are superconducting electrodes, magenta are normal metal traps, thin black interlayers are the tunnel barriers of NIS junctions. The incoming photons (orange waving arrows) generate rf-current in AlFe absorber. (**b**) Energy diagram of CEB. One photon is absorbed by one electron (top red ball), which redistributes its energy between other electrons in absorber N, changing the temperature of Fermi distribution. When voltage V is applied across the NIS junction, the hottest electrons tunnel into S (current I). After that, the electrons move away from the tunnel barrier and lose their energy in N trap. (**c**) Schematic representation of Andreev current suppression in AlFe due to the presence of Fe grains. Symbols “$*$” indicate defects and impurities in Al. Paths 1 (reflection) and 2 (Andreev reflection) are present in a typical normal metal. Paths 3 and 4 show that an electron spin is not constant, if the scattering on the magnetic grains happens, decreasing the probability of Andreev reflection.

## Samples and experiment description

The maximum responsivity of CEB depends on the absorber volume $V_N$ and its electron temperature $T_e$ as $S \sim 1 / (V_N T_e^4)$^21^, which makes it very important to decrease both $V_N$ and $T_e$. An entirely normal metal absorber such as Cu^33^ is fabricated on top of superconducting Al electrodes because the reliable tunnel barrier is much easier to make on top of Al by its oxidation. As a result, the Cu layer of a SINIS junction is always thicker than the Al electrodes due to technological requirements.

If the absorber is made of Al with suppressed superconductivity, it can be deposited as the first layer and can be made as thin as possible (Fig. 1a). It decreases the absorber volume, the electronic heat capacity, and the electron–phonon coupling, therefore improving sensitivity^23,26,27^. In Fig. 1b, the energy diagram illustrates the hot electron tunneling with its later relaxation in N traps, thus preventing the heat return into the absorber.

The two-particle Andreev current^34,35^ is one of the most serious factors limiting the electron cooling efficiency since it dissipates heat in the normal metal. In Fig. 1c, the key advantage of S/F nanoabsorber is illustrated by showing the typical trajectories of electrons/ holes in the normal metal. An electron reaches the NIS interface (paths 1–2) after being scattered and finally penetrates the superconductor as a Cooper pair due to Andreev reflection, and a hole is retro-reflected. The Fe sublayer creates the magnetic scattering to destroy time-reversal symmetry in loop 3–4 (Fig. 1c) with proper dephasing of electron and reflected hole, thus suppressing Andreev reflections and increasing the electron cooling efficiency. Therefore, instead of using external magnetic fields^36,37^, resulting in Abrikosov vortex formation^38^ and superconducting gap suppression, we utilize the internal mechanism of Andreev heating current suppression with a thin 0.7 nm Fe layer.

**Figure 2:**
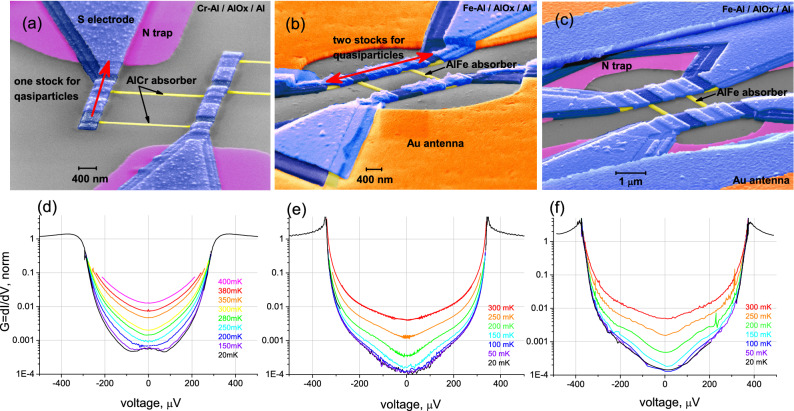
SEM images of the sample designs with one stock and normal metal traps for quasiparticles (**a**), with two stocks and without traps (**b**) and with two stocks and normal metal traps (**c**). Here the gold antennas are shown in orange, the superconductors in blue, the N traps in magenta, and the absorber in yellow, with a typical volume of its narrow part of $15\ \mathrm{nm}^{3} \times 80\ \mathrm{nm}^{3} \times 1000\ \mathrm{nm}^{3}$. Red arrows in (**a**,**b**) show the possible directions for quasiparticles to move. In (**a**), there is only one direction, which results in the S electrode overheating. Plots on (**d**,**e**,**f**) are differential conductivities of the samples **A**, **B**, and **C**, at various temperatures, respectively. In (**d**), the presence of Andreev current $I_{A}$ is evident below 150 mK as a slight peak at zero voltage. In (**e**,**f**), there is no visible sign of $I_{A}$ down to 20 mK. Much better electron cooling is seen in (**e**,**f**) in comparison with (**d**).

Three types of the samples, **A**, **B** and **C**, are shown in Fig. 2a–c with corresponding plots of differential conductivity d, e and f versus the voltage across the bolometer. In sample **A**, there is a Cr granular layer, and in the samples **B** and **C**, there is a Fe granular layer used to suppress the absorber’s superconductivity.

Sample **A** is an early version of the CEB. Even in this sample, the normal metal traps are implemented to prevent the heat return to the absorber. However, both SIN junctions are connected to the traps and the antenna from one side only (one stock for hot electrons). As a result, we have observed rather poor electron cooling due to overheating superconducting electrodes^26^. One can see also a zero bias peak on the conductivity plot (Fig. 2d), appearing at the temperature 150 mK and below, which is a clear signature of the Andreev current.

In sample **B**, we have significantly improved the absorber cooling by adding a second stock for hot quasiparticles to external superconducting electrodes at both SINIS ends^27,39^ (S electrodes contact the antenna from both sides of the absorber, compare with Fig. 2a). The normal metal traps are absent, but the thickness of the superconductive electrodes is increased, and the cooling efficiency of the samples with these modifications is already high enough, so only 6% of the removed heat returns back to the absorber^27^.

Sample **C** differs from **B** only by the added normal metal traps for quasiparticles under the superconducting electrodes. The experiments with these samples demonstrated that the return heat is just 0.5%. In other words, the electron cooling reaches its maximal efficiency.

For both samples **B** and **C** with a Fe underlayer in the absorber, the differential conductivity does not have a zero bias peak down to 20 mK (Fig. 2e,f). Thus, one can see that the Fe magnetic granular layer, with a thickness of just 0.7 nm, underneath the Al film, has changed the absorber properties significantly.

The parameters of the samples, which will be used for fitting in the next section, are listed in Table 1. All of them are measured experimentally, except for the returning power, which is determined from the solution of heat balance equations (HBE) (2), described below. Here $R_N$ is the normal resistance of the NIS junction, and $\sigma _{N}$ is the absorber electrical conductivity. It can be seen that the parameters of the tunnel junctions are quite similar for the three samples, while the differential conductivities are rather different. That means that the SINIS junctions themselves do not directly determine the cooling efficiency.

**Table 1:** Parameters of the samples.

Parameter	A	B	C
$R_N$ (1 NIS), k$\Omega$	0.7	1.6	1.25
$\sigma _{N}$, ${(\upmu \Omega \mathrm{cm})^{-1}}$	0.079	0.081	0.081
Volume of N, $\upmu \mathrm{m}^3$	0.02	0.02	0.02
Critical temperature, K	1.47	1.244	1.244
Area of NIS, $\upmu \mathrm{m}^2$	0.76	0.72	0.72
Returning power, %	30	6	0.5

## Electron temperature

The easiest way to find the electron temperature of the absorber is from the quasiparticle tunneling current:

1 $$\begin{aligned} I_{qp} = \int \limits _{-\infty }^{\infty } \frac{\nu (\varepsilon )}{e R_N}\left[ \frac{1}{\exp (\frac{\varepsilon -eV}{k_B T_e})+1} - \frac{1}{\exp (\frac{\varepsilon }{k_B T_s})+1}\right] d\varepsilon , \end{aligned}$$

where *V* is the voltage across the NIS junction, $T_e$ and $T_s$ are electron temperatures in the normal metal and the superconductor, respectively, $\nu (\varepsilon ) = \text {Re} [{\varepsilon } / {\sqrt{\varepsilon ^2 - \Delta ^ 2}}]$ is a density of states in the superconductor, $\Delta$ is a superconducting gap, $k_{B}$ is the Boltzmann constant. The current, the voltage, $R_N$ and $\Delta$ are measurable values, whereas $T_s$ can be set to the phonon temperature. Thus, the only unknown quantity $T_e$ in Eq. (1) can easily be extracted.

**Figure 3:**
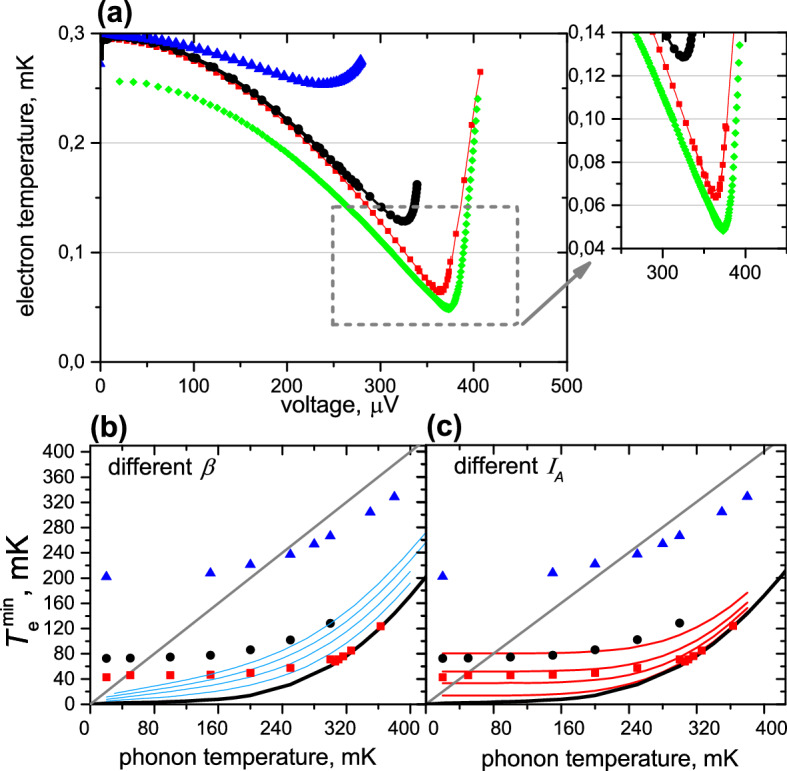
(**a**) Electron temperature obtained from HBE (2) versus voltage for three samples at $T_{ph}=300 mK$: triangles for the sample A, circles for the sample B, rectangles for the sample C. Diamonds are for the sample C at 256 mK. (**b**) Minimal electron temperature versus phonon temperature. Blue curves are calculated for various fixed $\beta$ (0.0,0.2,0.4 and 0.6) and zero $I_{A}$. (**c**) Minimal electron temperature versus phonon temperature. Red curves are calculated for various $I_{A}$ and zero $\beta$. $I_{A}$ changes from 30 pA to 4 nA. Thick black solid curve is an ideal theory without Andreev current and without heat return. In addition, experimental points are given: triangles for the sample A, circles for the sample B, rectangles for the sample C.

The electron temperatures of the three samples at the phonon temperature $T_{ph}=300 mK$ are shown in Fig. 3a versus the voltage across the bolometer. The sample **C** reaches the minimum electron temperature $T_e=65 mK$, which is close to a theoretical limit, predicted in^29^. One more curve for $T_{ph}=256 mK$ (green diamonds) is also given for sample **C** with minimum $T_e=48 mK$. Below 256 mK the minimum $T_e$ saturates at 42 mK for this sample. In Fig. 3b,c we plot the experimentally obtained minimum $T_e$ versus the phonon temperature for three of our samples, extracted with the help of Eq. (1).

The described method is fast, but not always precise, and gives very limited knowledge about the system. Therefore, we also use the heat balance equations (HBE) (2), see “Methods”. This allows estimating the contribution of each power flow channel separately. Namely this model helps us to find the best geometrical configuration of the CEB. Along with the cooling efficiency, HBE can be used to calculate the minimal electron temperature in an idealized system and to trace the influence of adverse factors. The HBE use is justified by very fast electron–electron interaction, which at low temperatures below 0.5K is much faster than the tunneling and the electron–phonon interaction, leading to quasi-equilibrium Fermi distribution of electrons in the absorber^13^.

In Fig. 3b,c, the theoretical minimum of $T_e$ (thick black curve), calculated for parameters of sample **C**, is plotted versus phonon temperature. In the calculation of the theoretical minimum, we disregard both the Andreev current $I_A$ and the return heat, i.e. $I_A=0$ and $\beta =0$. The return heat is characterized by a coefficient $0< \beta < 1$, which shows how much power, removed from the absorber, returns back (2). It is also shown here what happens if we add the nonzero return power from superconductor (Fig. 3b) or Andreev current (Fig. 3c) to the HBE. One can see that the two heating sources have different influence on minimum $T_e$. The return power $\beta$ increases minimum $T_e$ for all phonon temperatures, but does not lead to $T_e$ saturation at the lowest phonon temperatures. The Andreev current does not change the minimum $T_e$ at high phonon temperatures much, but gives a $T_e$ limit at low temperatures. $I_{A}$ does not transfer heat through the N/S interface, while generating the Joule heating $I_A V$ deposited in the N electrode^40^. That’s why the excess heating dominates single-particle cooling^6^ at low enough temperatures. In other words, at nonzero $\beta$ and $I_{A}=0$, the minimal electron temperature will be higher than the theoretical minimum in the whole range of phonon temperatures. But at $\beta =0$ and $I_{A}>0$, it is possible to reach the minimum theoretical electron temperature above a certain $T_{ph}$ value, determined by the value of Andreev current.

The minimum $T_e = 65 mK$ of sample **C** coincides with the theoretical curve at the temperature of 300 mK. Below this temperature, $T_e$ saturates at 42 mK and does not change anymore with the decrease of $T_{ph}$ due to the tiny Andreev current still persisting in our structure.

For sample **B**, one can see that only Andreev current cannot describe the dependence of the $T_e$ minimum on $T_{ph}$, therefore we also need to add a small $\beta$. We obtain that $\beta =0.06$ for this sample.

Sample **A** has rather poor cooling properties both due to high Andreev current and due to high $\beta =0.3$. From this, we conclude that the underlayer of Fe below Al suppresses the Andreev current more efficiently in comparison with Al/Cr system.

**Figure 4:**
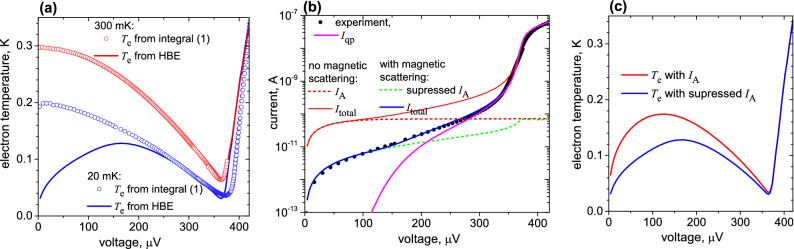
The fitting results for the sample **C**. (**a**) The electron temperature determined by two different methods: symbols are for using integral for quasiparticle tunneling (1), lines for using HBE (2) and (3), blue and red colors are for plate temperatures 20 mK and 300 mK, respectively. (**b**) Current–voltage characteristic at 20 mK. Dots represent an experiment, and magenta is quasiparticle current. The red solid curve shows the total current if Andreev current (dashed red curve) is not suppressed. The blue solid curve is the total current with suppressed Andreev current (dashed green). (**c**) Electron temperature versus voltage at $T_{ph}=20 mK$ in case of suppressed and non-suppressed Andreev current.

Below, we obtain the electron temperatures by two different methods: by Eq. (1) and HBE (2) with an account of Andreev current (3) (see “Methods”). Let us show that Eq. (1) works well if a leakage current or $I_A$ are negligible compared to a quasiparticle current. In Fig. 4a, we show the electron temperature, obtained from Eq. (1) and the HBE (2), at two temperatures, 300 mK and 20 mK. One can see that both methods give the same results at 300 mK. But at 20 mK, the results are rather different. Eq. (1) overestimates the electron temperature at low voltages because it does not consider the two-particle current $I_A$. At the same time, the minimum electron temperature near the gap has very similar values for both methods though at slightly different voltages.

**Figure 5:**
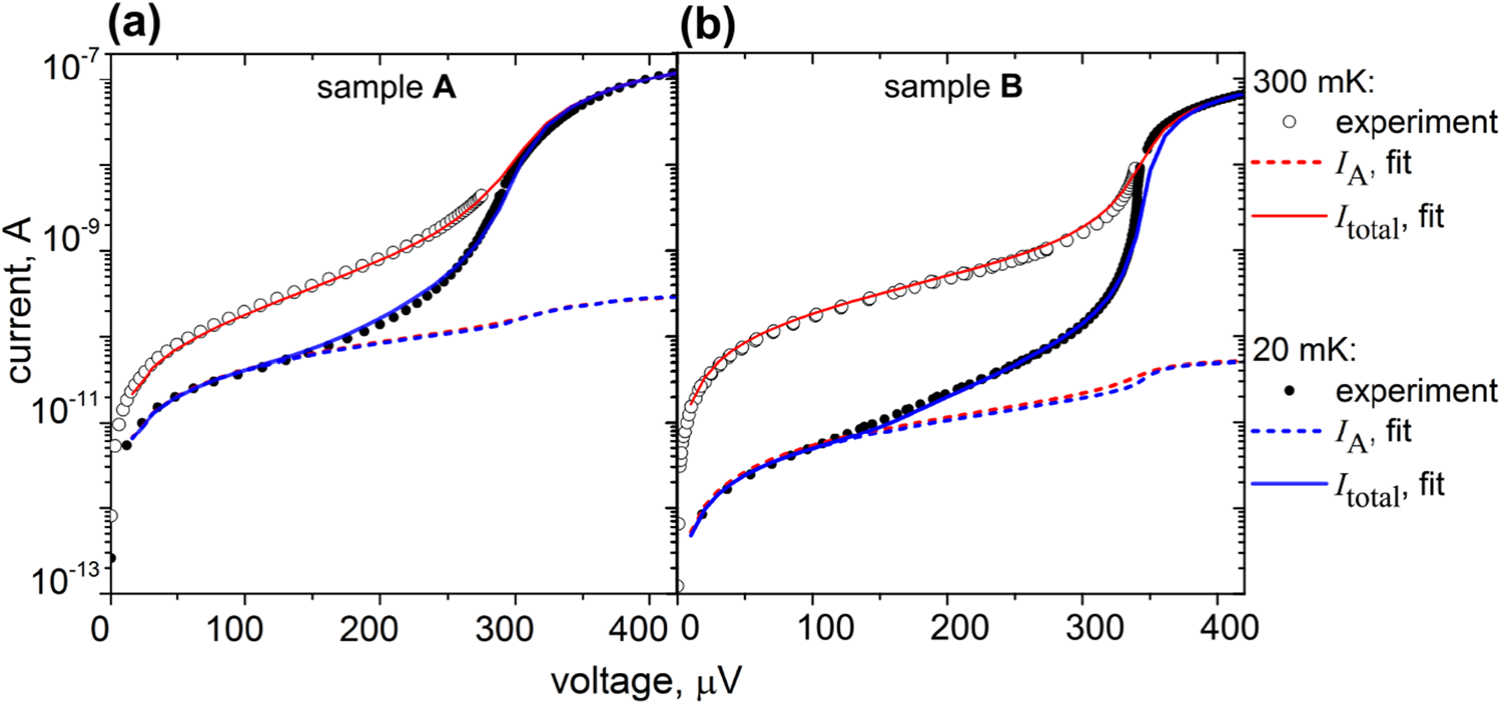
The fitting of IV-curves by HBE with Andreev current. (**a**) The sample **A**. (**b**) The sample **B**. The color legend from the right is valid for both (**a**,**b**) blue color is for 20 mK, red color is for 300 mK, dots are an experiment, solid curves are the total fitted current, the dashed curve shows Andreev current.

The results of IV-curves fitting for the samples **A**, **B**, **C** using the HBE model are shown in Figs. 4 and 5 for 20 mK and 300 mK. The heat balance equation (2), supplemented by the Andreev current (3) and heating from it, rather accurately describes the experimental data and does not need additional fitting effects, such as leakage current or gap smearing due to environment-assisted tunneling^41^. The IV-curves agree with the experiment at the proper value of the magnetic scattering parameter $\tau _m$ (5) (see “Methods”). The quasiparticle current, shown by the blue curve, fits the experimental curve well near the gap, but gives too small current at low voltages. The fit becomes much closer to the experiment if we add Andreev current to the model and take into account the heat $P_{A}=I_A V$, dissipated in the normal absorber.

In sample **A**, we have an underlayer of Cr with a thickness 0.7 nm, which is also ferromagnetic and could suppress the Andreev current similar to Fe. Indeed, in fitting, we obtain some suppression of Andreev current for this sample, but the effect is weaker than for the samples with Fe.

By comparing the fit of samples **A** and **B**, shown in Fig. 5a,b, respectively, one can see that the Andreev current is approximately one order of magnitude larger for the sample **A** than for **B**. We assume that this difference is due to both higher barrier transparency for sample **A** (a factor of two), but mostly because of the Fe underlayer in sample **B**.

## Discussion

Let us discuss several limitations of cooling performance and how to overcome them to get high efficiency of electron cooling. The first limitation arises due to the accumulation of nonequilibrium quasiparticles injected into the S layer near the NIS interface^42,43^. The consequences are the heat back current from hot quasiparticles via phonons in superconductor and substrate to the absorber, and the overheating of the superconducting electrode leading to the gap suppression. Despite the difficulty of theoretical analysis with an account of nonequilibrium effects, there is a practical solution to remove hot quasiparticles from the superconducting electrode by the traps^44^ made of an additional normal metal layer covering the S layer. The second limitation arises from the intrinsic multiparticle nature of current transport in NIS junctions consisting not only of single-particle tunneling but also of two-particle Andreev tunneling^45,46^. The single-particle current and the associated heat current, formed by quasiparticles with energies larger than the superconducting gap, are exponentially suppressed in the sub-gap voltage region at low temperatures. The charge is mainly transferred by means of Andreev reflections of quasiparticles with sub-gap energies^34^, dissipating the heat in the normal metal electrode. Thus, the interplay between the single-particle tunneling and Andreev reflections sets a limiting temperature for the refrigeration, depending on the interface transparency^47^.

A strong reduction of the Andreev current is anticipated in materials, in which the proximity effect is suppressed. Indeed, for this reason the electron cooling efficiency improving was theoretically predicted in the case of ferromagnetic interlayers^48–50^, or a spin-filter barriers^29^ in SINIS structures. However, there were no experimental observations of this effect so far. On the contrary, very significant excess conductivity, by a factor of three exceeding the minimal $\mathrm {d}I/\mathrm {d}V$ value, was demonstrated for Cu suspended absorber in^33^.

The suppression of the excess conductance due to Andreev reflections can be done by an external magnetic field^36,37^. However, it leads, for example, to Abrikosov vortex formation^38^ and to superconducting gap suppression. Instead, we utilize the internal mechanism of Andreev current suppression, provided by a thin 0.7 nm Fe layer. It does not affect the superconducting gap of S lead, since the S electrodes are protected by the tunnel barrier, but improves the electron cooling^29^.

In this work, we have overcome both limitations and demonstrate the theoretical minimum of the electron temperature experimentally down to 65 mK in SINIS structures at 300 mK phonon temperature. This is an important threshold temperature because it can be reached in $^3$He cryostats. We also show electron cooling from 256 mK (which can be reached in two-stage $^3$He cryostats) to 48 mK. The suppression of excessive heating was achieved due to the following: the implementation of the hybrid S/F nanoabsorber instead of a normal metal nanoabsorber, the modification of the tunnel junctions electrodes geometry and the addition of specially designed normal metal traps for non-equilibrium quasiparticles. In contrast to the external magnetic field, the internal mechanism by ferromagnetic sublayer is performing two-particle Andreev current suppression in a delicate manner without the S electrodes superconducting gap suppression. Simultaneously, the suppression of two-particle tunneling decreases the shot noise^51^. The deep electron cooling demonstrated in this paper gives these CEBs record sensitivity that makes them promising for receivers on prospective future space missions. In addition, the possibility to suppress the heat transfer due to a two-particle current is the next step for reliable quantum caloritronics^14,52,53^.

## Online content

Any methods, additional references, Nature Research reporting summaries, source data, statements of code and data availability and associated accession codes are available upon reasonable request from L. S. K.

## Methods

### Sample fabrication

All the samples studied in this work were fabricated at Chalmers University of Technology. For samples A and C, the manufacturing technology consisted of three stages, for sample B is was of two stages, due to the absence of normal metal traps. All layers, except for the bolometers themselves, were formed by the method of lift-off lithography, performed using a laser-writer, and the subsequent deposition of thin films by an electron beam. For the fabrication of bolometers, an electronic lithograph and a shadow evaporation technique were used, making it possible to deposit tunnel junctions without breaking the vacuum. Normal metal traps, such as in sample C, were made of three metals: 1 nm of titanium, 15 nm of gold and 2 nm of palladium. Antennas were made of the same metals, but of increased thickness: 10 nm of titanium, 120 nm of gold and 20 nm of palladium. The bolometers represent SINIS structures, made by the self-aligned shadow evaporation technique. The layer of normal metal is deposited first and is made of two thin films: 0.7 nm of Cr/CrOx and 14 nm of Al for the sample **A**, or 0.7 nm of Fe and 14 nm of Al for the samples **B** and **C**. The thin layer of Cr or Fe below Al is needed to suppress the superconductivity in the absorber. After that, the aluminum with suppressed superconductivity is oxidized, and the electrodes from superconducting aluminum are deposited at two different angles.

### Heat balance equation

The electron temperature can be obtained from the IV-curve, using the integral for the tunneling current through a NIS junction (1). It gives a reliable result if the total current is composed only of one-particle component. Otherwise, we have to use more a complicated approach, based on the heat balance equation (HBE)^26,27^:

2 $$\begin{aligned} P_N + P_{ph-e} - 2 P_{cool} + 2\beta P_S + P_{A} = 0. \end{aligned}$$

In Eq. (2), $P_N$ is the Joule heat in the N absorber, $P_{ph-e} = \Sigma V_N (T^5_{ph} - T^5_e)$ is a heat flow between electron and phonon subsystems, $\Sigma$ is an electron–phonon coupling constant and $V_N$ is the volume of the N absorber. $P_{cool}$ is a cooling power of NIS junction, $P_S$ is the net power dissipated in the S electrode, and coefficient $\beta$ shows how much of $P_S$ returns back to the N absorber, and $P_{A} = I_{A}V$ is the heating due to the Andreev current, *V* is the voltage drop across the NIS junction.

For the planar geometry of the junction at $0< \varepsilon < \Delta$, we get that Andreev current is expressed as^47^

3 $$\begin{aligned} I_A= & {} -\frac{1}{2e R_N} \int \limits _{0}^{\Delta } \frac{\Delta d\varepsilon }{\sqrt{{\Delta }^2 - \varepsilon ^2}} \mathrm {Im}(\theta _0)\cdot {}\nonumber \\&\cdot \left[ \tanh \left( \frac{\varepsilon +eV}{2 k_B T_e}\right) - \tanh \left( \frac{\varepsilon -eV}{2 k_B T_e}\right) \right] . \end{aligned}$$

The parametrized Green function was calculated using Uzadel equation with Kupriyanov–Lukichev boundary conditions^54^

4 $$\begin{aligned} \theta _0 = \frac{2 W \Delta }{-i k^2\xi _0^2\sqrt{\Delta ^2 - \varepsilon ^2} + 2 W \varepsilon }, \end{aligned}$$

taking into account the decay of the state with the wave vector *k* due to spin scattering

5 $$\begin{aligned} k\xi _0 = \sqrt{\frac{\varepsilon + i/\tau _m}{i\Delta }}. \end{aligned}$$

Here, the parameter of magnetic scattering $\tau _m$ is to be found from fitting, $W = W_0 \xi _0/d$ is the effective tunneling parameter for planar tunnel junctions, used in our CEB, with $W_0 = R(\xi _0)/R_N$, the standard tunnelling parameter^47^. For aluminium $\xi _0 = 100 \mathrm{nm}$ and in our samples $d = 14 \mathrm{nm}$. $R_N$ is the normal resistance of the junction, $R(\xi _0)$ is the resistance of Al/Fe absorber of the length $\xi _0$. Then we get $W_0 \sim 10^{-5}$ and $W \sim 10^{-4}$. The fitting parameters related to Andreev current for the sample **A** are $W = 1.5 \times 10^{-4}$ and $\tau _m = {1}$, for the sample **B**, they are $W = 0.7\times 10^{-4}$ and $\tau _m = {0.5}$, for the sample **C**, we have $W = 0.9\times 10^{-4}$ and $\tau _m = {0.5}$.

In Fig. 4, the results of $T_e$ calculation from Eq. (1) and from the HBE (2) at 300 mK and 20 mK are shown. Both methods give the same results at 300 mK, but differ at 20 mK, while the minimum electron temperature has very similar values for both methods, though at slightly different voltages.

## Data Availability

The data that support the plots within this paper and other findings of this study are available from the corresponding author upon reasonable request.

## References

[1] Birkinshaw M (1999). The Sunyaev–Zel’dovich effect. Phys. Rep..

[2] Hand E (2009). Cosmology: The test of inflation. Nature.

[3] Planck and BICEP2/Keck coll (2015). Joint analysis of BICEP2/Keck array and planck data. Phys. Rev. Lett..

[4] Kokkoniemi R (2019). Nanobolometer with ultralow noise equivalent power. Commun. Phys..

[5] Nguyen HQ, Meschke M, Courtois H, Pekola JP (2014). Sub-50-mK electronic cooling with large-area superconducting tunnel junctions. Phys. Rev. Appl..

[6] Rajauria S (2008). Andreev current-induced dissipation in a hybrid superconducting tunnel junction. Phys. Rev. Lett..

[7] Triqueneaux S, Sentis L, Camus Ph, Benoit A, Guyot G (2006). Design and performance of the dilution cooler system for the Planck mission. Cryogenics.

[8] Holmes W (2010). Sub-Kelvin cooler configuration study for the background limited infrared submillimeter spectrometer BLISS on SPICA. Cryogenics.

[9] Chaudhry G, Volpe A, Camus P, Triqueneaux S, Vermeulen G (2012). A closed-cycle dilution refrigerator for space applications. Cryogenics.

[10] Nahum M, Eiles TM, Martinis JM (1994). Electronic microrefrigerator based on a normal-insulator–superconductor tunnel junction. Appl. Phys. Lett..

[11] Giazotto F, Heikkilä TT, Luukanen A, Savin AM, Pekola JP (2006). Opportunities for mesoscopics in thermometry and refrigeration: Physics and applications. Rev. Mod. Phys..

[12] Muhonen JT, Meschke M, Pekola JP (2012). Micrometre-scale refrigerators. Rep. Prog. Phys..

[13] Pekola JP (2015). Towards quantum thermodynamics in electronic circuits. Nat. Phys..

[14] Fornieri A, Giazotto F (2017). Towards phase-coherent caloritronics in superconducting circuits. Nat. Nanotechnol..

[15] Pekola JP (2004). Limitations in cooling electrons using normal-metal-superconductor tunnel junctions. Phys. Rev. Lett..

[16] Clark AM (2005). Cooling of bulk material by electron-tunneling refrigerators. Appl. Phys. Lett..

[17] O’Neil GC, Lowell PJ, Underwood JM, Ullom JN (2012). Measurement and modeling of a large-area normal-metal/insulator/superconductor refrigerator with improved cooling. Phys. Rev. B.

[18] Lowell PJ, O’Neil GC, Underwood JM, Ullom JN (2013). Macroscale refrigeration by nanoscale electron transport. Appl. Phys. Lett..

[19] Pekola JP (2000). Microrefrigeration by quasiparticle tunnelling in NIS and SIS junctions. Phys. B.

[20] Wellstood FC, Urbina C, Clarke J (1994). Hot-electron effects in metals. Phys. Rev. B.

[21] Kuzmin, L. Optimization of the hot-electron bolometer and a cascade quasiparticle amplifier for space astronomy. In *International Workshop on Superconducting Nano-Electronics Devices* **145–154**. (Springer, Boston, 2002). 10.1007/978-1-4615-0737-6_16.

[22] Kuzmin, L. Ultimate cold-electron bolometer with strong electrothermal feedback. In *Proceedings of SPIE: Millimeter and Submillimeter Detectors for Astronomy II* Vol. 5498 (eds Zmuidzinas, J. *et al.*) 349–361 (International Society for Optics and Photonics (SPIE), 2004). 10.1117/12.554317.

[23] Tarasov MA, Kuzmin LS, Edelman VS, Mahashabde S, de Bernardis P (2011). Optical response of a cold-electron bolometer array integrated in a 345-GHz cross-slot antenna. IEEE Trans. Appl. Supercond..

[24] Brien TLR (2014). A strained silicon cold electron bolometer using Schottky contacts. Appl. Phys. Lett..

[25] Brien TLR (2016). Optical response of strained- and unstrained-silicon cold-electron bolometers. J. Low Temp. Phys..

[26] Gordeeva AV (2017). Observation of photon noise by cold-electron bolometers. Appl. Phys. Lett..

[27] Kuzmin LS (2019). Photon-noise-limited cold-electron bolometer based on strong electron self-cooling for high-performance cosmology missions. Commun. Phys..

[28] Leivo MM, Pekola JP, Averin DV (1996). Efficient Peltier refrigeration by a pair of normal metal/insulator/superconductor junctions. Appl. Phys. Lett..

[29] Kawabata S, Ozaeta A, Vasenko AS, Hekking FWJ, Bergeret FS (2013). Efficient electron refrigeration using superconductor/spin-filter devices. Appl. Phys. Lett..

[30] Flokstra MG (2016). Remotely induced magnetism in a normal metal using a superconducting spin-valve. Nat. Phys..

[31] Kuzmin L, Agulo I, Fominsky M, Savin A, Tarasov M (2004). Optimization of electron cooling by SIN tunnel junctions. Supercond. Sci. Technol..

[32] Rajauria S (2007). Electron and phonon cooling in a superconductor-normal-metal-superconductor tunnel junction. Phys. Rev. Lett..

[33] Tarasov M (2017). Electrical and optical properties of a bolometer with a suspended absorber and tunneling-current thermometers. Appl. Phys. Lett..

[34] Andreev AF (1964). The thermal conductivity of the intermediate state in superconductors. J. Exp. Theor. Phys..

[35] Yeyati AL, Bergeret FS, Martín-Rodero A, Klapwijk TM (2007). Entangled Andreev pairs and collective excitations in nanoscale superconductors. Nat. Phys..

[36] Kastalsky A (1991). Observation of pair currents in superconductor–semiconductor contacts. Phys. Rev. Lett..

[37] van Wees BJ, de Vries P, Magnée P, Klapwijk TM (1992). Excess conductance of superconductor–semiconductor interfaces due to phase conjugation between electrons and holes. Phys. Rev. Lett..

[38] Arutyunov KYu, Suppula TI, Suoknuuti JK, Pekola JP (2000). Influence of magnetic field on cooling by normal-insulator–superconductor junctions. J. Appl. Phys..

[39] Kuzmin, L. S. *et al.* Realization of cold-electron bolometers with ultimate sensitivity due to strong electron self-cooling. In *IEEE Proceedings: 2017 16th International Superconductive Electronics Conference (ISEC)*, 1–4. 10.1109/ISEC.2017.8314194 (IEEE, 2018).

[40] Bardas A, Averin D (1995). Peltier effect in normal-metal-superconductor microcontacts. Phys. Rev. B.

[41] Pekola JP (2010). Environment-assisted tunneling as an origin of the dynes density of states. Phys. Rev. Lett..

[42] Rajauria S, Courtois H, Pannetier B (2009). Quasiparticle-diffusion-based heating in superconductor tunneling microcoolers. Phys. Rev. B.

[43] Vasenko AS, Hekking FWJ (2009). Nonequilibrium electron cooling by NIS tunnel junctions. J. Low Temp. Phys..

[44] Pekola JP (2000). Trapping of quasiparticles of a nonequilibrium superconductor. Appl. Phys. Lett..

[45] Hekking FWJ, Nazarov YuV (1993). Interference of two electrons entering a superconductor. Phys. Rev. Lett..

[46] Hekking FWJ, Nazarov YuV (1994). Subgap conductivity of a superconductor-normal-metal tunnel interface. Phys. Rev. B.

[47] Vasenko AS, Bezuglyi EV, Courtois H, Hekking FWJ (2010). Electron cooling by diffusive normal metal-superconductor tunnel junctions. Phys. Rev. B.

[48] Giazotto F, Taddei F, Fazio R, Beltram F (2002). Ultraefficient cooling in ferromagnet–superconductor microrefrigerators. Appl. Phys. Lett..

[49] Ozaeta A, Vasenko AS, Hekking FWJ, Bergeret FS (2012). Electron cooling in diffusive normal metal-superconductor tunnel junctions with a spin-valve ferromagnetic interlayer. Phys. Rev. B.

[50] Ozaeta A, Vasenko AS, Hekking FWJ, Bergeret FS (2012). Andreev current enhancement and subgap conductance of superconducting SFN hybrid structures in the presence of a small spin-splitting magnetic field. Phys. Rev. B.

[51] Wei J, Chandrasekhar V (2010). Positive noise cross-correlation in hybrid superconducting and normal-metal three-terminal devices. Nat. Phys..

[52] De Franceschi S, Mingo N (2007). Cooling electrons one by one. Nat. Nanotechnol..

[53] Saira O-P (2007). Heat transistor: Demonstration of gate-controlled electronic refrigeration. Phys. Rev. Lett..

[54] Kuprianov MY, Lukichev VF (1988). Influence of boundary transparency on the critical current of "dirty" ss’s structures. J. Exp. Theoret. Phys..

